# Graphene Oxide and Oxidized Carbon Black as Catalyst for Crosslinking of Phenolic Resins

**DOI:** 10.3390/polym11081330

**Published:** 2019-08-10

**Authors:** Maria Rosaria Acocella, Aniello Vittore, Mario Maggio, Gaetano Guerra, Luca Giannini, Luciano Tadiello

**Affiliations:** 1 Department of Chemistry and Biology and INSTM Research Unit, Università di Salerno, 84084 Fisciano (SA), Italy; 2 Pirelli Tyre SpA, Viale Sarca 222, 20126 Milano, Italy

**Keywords:** X-ray diffraction, DSC, FTIR, resorcinol, hexa(methoxymethyl)melamine

## Abstract

Influence of different graphite-based nanofillers on crosslinking reaction of resorcinol, as induced by hexa(methoxymethyl)melamine, is studied. Curing reactions leading from low molecular mass compounds to crosslinked insoluble networks are studied by indirect methods based on Differential Scanning Calorimetry. Reported results show a catalytic activity of graphene oxide (eGO) on this reaction, comparable to that one already described in the literature for curing of benzoxazine. For instance, for an eGO content of 2 wt %, the exothermic crosslinking DSC peak (upon heating at 10 °C/min) shifted 6 °C. More relevantly, oxidized carbon black (oCB) is much more effective as catalyst of the considered curing reaction. In fact, for an oCB content of 2 wt %, the crosslinking DSC peak can be shifted more than 30 °C and a nearly complete crosslinking is already achieved by thermal treatment at 120 °C. The possible origin of the higher catalytic activity of oCB with respect to eGO is discussed.

## 1. Introduction

Polymer nanocomposites have attracted a great interest from researchers, due to significant increase of properties obtained with low amounts of nano-dispersed fillers. Nanographite and graphene nanoplatelets have been largely studied as fillers for polymer nanocomposites [1,2,3,4,5,6,7,8,9,10,11]. Graphene is generally dispersed in various polymer matrices as reduced graphene oxide. Polymer matrices reinforced with graphene and nano-graphite platelets present higher electrical and thermal conductivity, improved strength, modulus, heat distortion temperature and barrier properties.

Many studies have been devoted to nanocomposites with epoxy matrices and graphite nanofillers. Some of these studies have shown a catalytic activity of graphene oxide and of oxidized carbon black on the reaction between the epoxy and amine groups of the resin, leading to higher crosslinking density in milder conditions [12,13,14,15,16,17,18,19,20]. This catalytic activity, suggested by the well-established catalytic behavior of graphene oxide for many organic reactions [21,22,23,24,25,26,27,28,29,30,31,32], has been proved by experiments on the epoxide ring opening reaction, for monofunctional epoxide and amine reactants, which lead to an uncrosslinked product being suitable for full chemical characterization [17,19].

Many studies have been also devoted to nanocomposites with graphite nanofillers in different kinds of phenolic resins [33,34,35,36,37,38,39,40,41]. One of these studies [39] has clearly shown that reduced graphene oxide (r-GO) can enhance mechanical properties and thermal stability of composites. Moreover, a study on benzoxazine-based composites has shown that addition of a few weight percent of graphene oxide accelerates the ring opening reactions leading to not only a decrease in cure temperature, but also to improvements of thermal stability [42].

**Figure 1:**
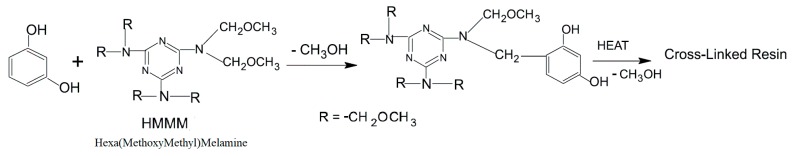
Scheme of the crosslinking reaction of the used phenolic resin.

In the present paper, the possible catalytic activity of graphene oxide and of oxidized carbon black on the crosslinking reaction of resorcinol, as induced by hexa(methoxymethyl)melamine (HMMM), is investigated (Figure 1). As in previous reports [16,19,42], the kinetic studies are mainly based on isothermal and non-isothermal scans by Differential Scanning Calorimetry.

## 2. Experimental

### 2.1. Materials

The hexa(methoxymethyl)melamine (HMMM) was Cyrez 963 from Allnex (Frankfurt am Main, Germany) and resorcinol was Rhenogran Resorcinol 80 from Rhein Chemie (Mannheim, Germany).

Synthetic Graphite 8427^®^ with a high surface area (HSAG, of about 308 m^2^/g) and a high shape anisotropy of the crystallites [41] was purchased from Asbury Graphite Mills Inc (Asbury, NJ, USA).

Two different carbon black samples, with designated grades N110 and N234 according to the American Society for Testing and Materials (ASTM)/D1765, and exhibiting BET surface areas of 151 m^2^/g and 125 m^2^/g, respectively, were purchased from Cabot (Alpharetta, GA, USA).

All other standard reagents were bought from Aldrich. 

### 2.2. Procedures

#### Preparation of GO and oCB Samples

Graphite oxide (GO) and oxidized carbon black (oCB) were prepared by Hummers’ method [43]. A total of 120 mL of sulfuric acid and 2.5 g of sodium nitrate were introduced into a 2000 mL three-neck round-bottomed flask immersed into an ice bath and 5 g of carbon samples were added, with magnetic stirring. After obtaining a uniform dispersion, 15 g of potassium permanganate were added very slowly to minimize the risk of explosion. The reaction mixture was thus heated to 35 °C and stirred for 24 h. Deionized water (700 mL) was added in small amounts into the resulting black and dark green slurry for carbon black and graphite, respectively, under stirring and, finally, gradually adding 5 mL of H_2_O_2_ (30 wt %). The obtained sample was poured into 7 L of deionized water, and then centrifuged at 10,000 rpm for 15 min with a Hermle Z 323 K centrifuge (Wehingen, Germany). The isolated GO and oCB powders were first washed twice with 100 mL of a 5 wt % HCl aqueous solution and subsequently washed with 500 mL of deionized water. Finally, powders were dried at 60 °C for 12 h. About 6 g of oCB and 7 g of GO powders were obtained. 

Exfoliated graphite oxide (eGO) samples were prepared by GO powders introduced in 125 mL ceramic jars (inner diameter of 75 mm) together with stainless steel balls (10 mm in diameter) and dry-milled in a planetary ball mill Fritsch Pulverisette 7 for 2 h with a milling speed of 500 rpm and a ball-to-powder mass ratio of 10 to 1.

Carbon nanofillers and resorcinol were dispersed in HMMM by mechanical stirring.

### 2.3. Characterization Techniques

#### 2.3.1. Elemental Analysis

Elemental analyses were conducted by a Thermo FlashEA 1112 Series CHNS-O analyzer (Waltham, MA, USA). The analyzed samples were pretreated in an oven for 12 h at 100 °C.

Elemental analysis of the obtained graphite oxide, graphene oxide and oxidized carbon black samples are reported in Table 1.

#### 2.3.2. Wide-Angle X-ray Diffraction

Wide-angle X-ray diffraction (WAXD) patterns were obtained by an automatic Bruker D8 Advance diffractometer (Karlsruhe, Germany), in reflection, at 35 KV and 40 mA, using nickel filtered Cu-Kα radiation (1.5418 Å). The *d*-spacings were calculated using Bragg’s law and the observed integral breadths (*β*obs) were determined by a fit with a Lorentzian function of the intensity corrected diffraction patterns. The instrumental broadening (*β*inst) was also determined by fitting of Lorentzian function to line profiles of a standard silicon powder 325 mesh (99%). For each observed reflection, the corrected integral breadths were determined by subtracting the instrumental broadening of the closest silicon reflection from the observed integral breadths, *β* = *β*obs − *β*inst. The correlation lengths (*D*) were determined using Scherrer’s equation:

(1) $$D=\frac{K\lambda }{\beta \cos\theta }$$

where *λ* is the wavelength of the incident X-rays and 2*θ* the diffraction angle, assuming the Scherrer constant *K* = 1.

#### 2.3.3. FTIR Spectra

FTIR spectra were obtained with a FTIR (BRUKER Vertex70, Ettlingen, Germany) spectrometer equipped with a deuterated triglycine sulfate (DTGS) detector and a KBr beam splitter, at a resolution of 2.0 cm^−1^. The frequency scale was internally calibrated to 0.01 cm^−1^ using a He-Ne laser. A total of 32 scans were signal averaged to reduce the noise. Spectra of powder samples were collected by using potassium bromide (KBr) pellets.

#### 2.3.4. Differential Scanning Calorimetry

The reactivity of filled and unfilled phenolic resin was measured using a differential scanning calorimeter (TA instruments DSC Q2000, New Castle, DE, USA).

## 3. Results

### 3.1. X-ray Diffraction and FTIR Characterizations of the Graphite-Based Nanofillers

**Figure 2:**
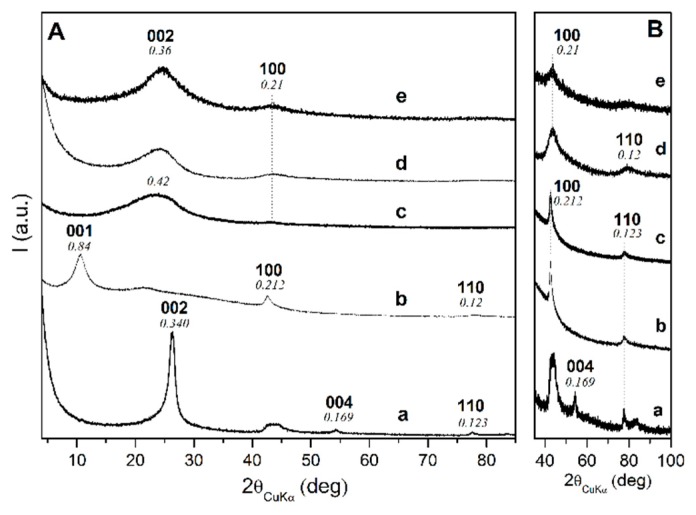
X-ray diffraction patterns (Cu Kα) of the high-surface-area graphite (HSAG) (**a**), of the derived GO (**b**) and eGO (**c**), and of one of the used carbon black, CB N110 (**d**) and of the corresponding oxidized sample (**e**).

X-ray diffraction patterns of the graphite-based fillers used in the present study are reported in Figure 2. The used high-surface-area graphite (HSAG with a negligible oxygen content, Figure 2a) shows an interlayer distance of 0.34 nm, and a high shape anisotropy (*d*_║_/*d*_┴_ = 3.1) [44]. The X-ray diffraction pattern of the derived graphite oxide (GO, with an O/C weight ratio of 0.71) shows an increase of the interlayer distance from 0.34 nm to 0.84 nm (Figure 2b). The correlation length perpendicular to the layers (as evaluated from the first 00*l* reflection) decreases from 9.8 nm to 4.2 nm, while the in-plane correlation length (as evaluated from the 100 reflection) remains almost unchanged (*d*_║_ ≈ 30 nm), thus leading to an increased shape anisotropy up to *d*_║_/*d*_┴_ = 7. The X-ray diffraction pattern of graphene oxide (eGO, Figure 2c), as derived by ball-milling of graphite oxide shows the complete disappearance of the 001 and 004 reflections and maintenance of 100 and 110 reflections, confirming the maintenance of in-plane crystalline order and complete loss of crystalline order perpendicular to the graphitic planes, i.e., a complete graphite oxide exfoliation. The WAXD pattern of one of the considered oCB samples (CB N110) and of the corresponding oxidized sample are shown in Figure 2d,e, respectively. As discussed in detail in Reference [45], these patterns can be interpreted as a disordered spatial arrangement of graphene and graphene oxide layers, exhibiting short in-plane correlation lengths. For instance, the in-plane correlation length, as evaluated on the basis of the half-height-width of the 100 reflections in Equation (1), is equal to 26 nm and 4 nm for the graphene oxide and oxidized oCB samples of Figure 2c,e, respectively.

**Figure 3:**
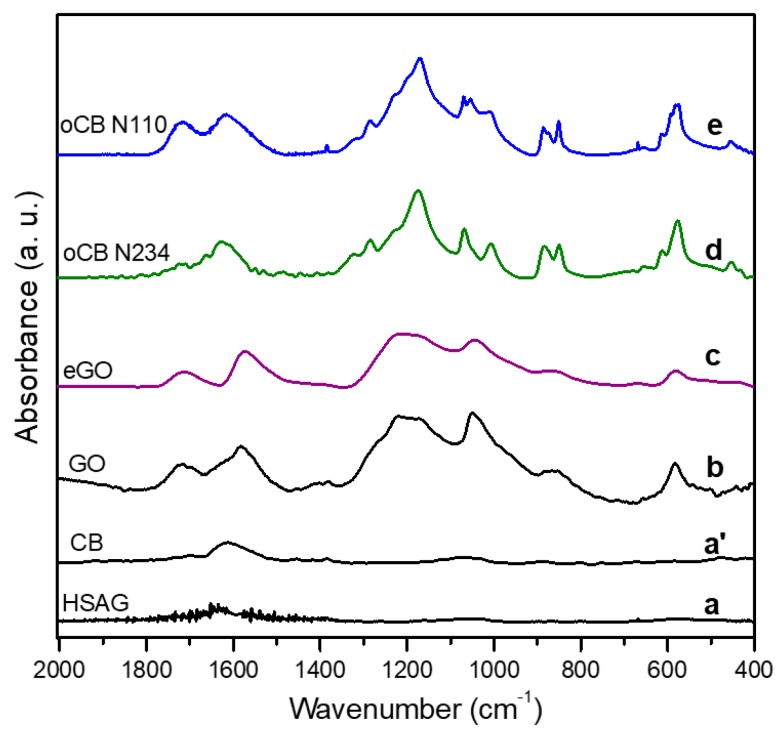
FTIR spectra, in the wavenumber range 2000–400 cm^−1^, of the high-surface-area graphite (HSAG) (**a**), of the derived GO (**b**) and eGO (**c**), and of the oxidized carbon black samples, oCB N234 (**d**) and oCB N110 (**e**).

FTIR spectra of the considered carbon materials are collected in Figure 3. It is apparent that the spectra of the oCB samples (Figure 3d,e) are much better resolved than the spectra of graphite oxide (Figure 3b) and of graphene oxide (Figure 3c). This is, of course, related to their much shorter in-plane correlation length, i.e., much smaller lateral size of the oxidized graphene layers, as pointed out by the WAXD patterns of Figure 2.

### 3.2. DSC Study of Crosslinking of the Phenolic Resin

**Figure 4:**
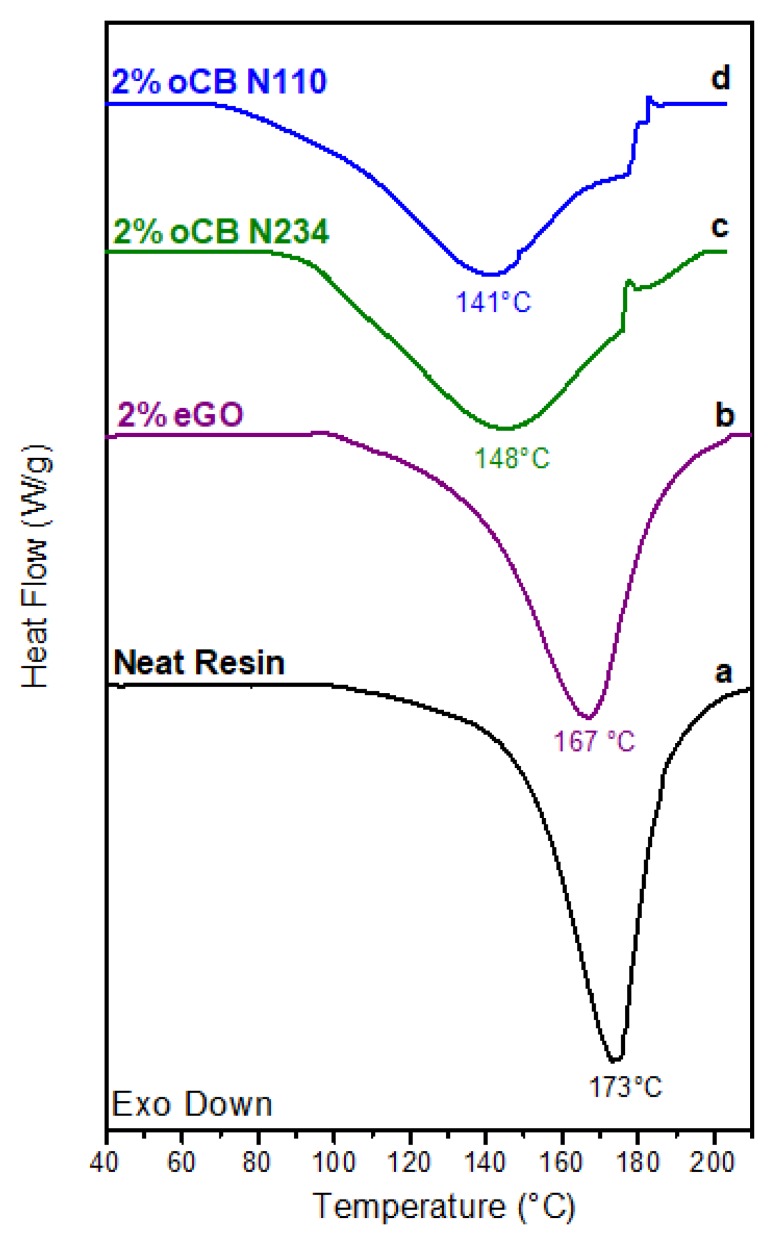
DSC scans at a heating rate of 10 °C/min for resorcinol/HMMM resins (for the weight ratio 1): (**a**) without filler; (**b**–**d**) with 2 wt % of carbon filler: (**b**) eGO; (**c**) oCB-N234; and (**d**) oCB-N110.

The DSC scan of neat resin with a weight ratio resorcinol/HMMM equal to 1 is shown in Figure 4a. The scan shows a crosslinking exothermic peak with an enthalpy change of nearly 270 J/g whose maximum is located at 173 °C.

The addition to the mixture of 2 wt % of GO affects the position of the exothermic peak, whose maximum shifts down to 167 °C (Figure 4b). An analogous behavior has been described in the literature for curing of benzoxazine, whose crosslinking peak shifts from 261 °C to 250 °C, due to an addition to the resin of 1 wt % of GO [42]. The influence of oCB on the studied crosslinking reaction is much stronger. In fact, as shown for two oCB samples, the exothermic peaks maintain nearly the same area but become much broader and are largely shifted toward lower temperatures (Figure 4c,d). In particular, for the oCB samples with an O/C ratio and S content similar to those of the used eGO sample, the peak maximum moves down to 148 °C (Figure 4c). The exothermic peak is further moved to 141 °C, by using the oCB sample with a higher O/C weight ratio (Figure 4d).

It is worth noting the presence of small superimposed endothermic peaks, roughly located at 175 °C (Figure 4c,d), typical of oxidized carbon black, as for instance shown by DSC scans of Figure 3 of Reference [45].

**Figure 5:**
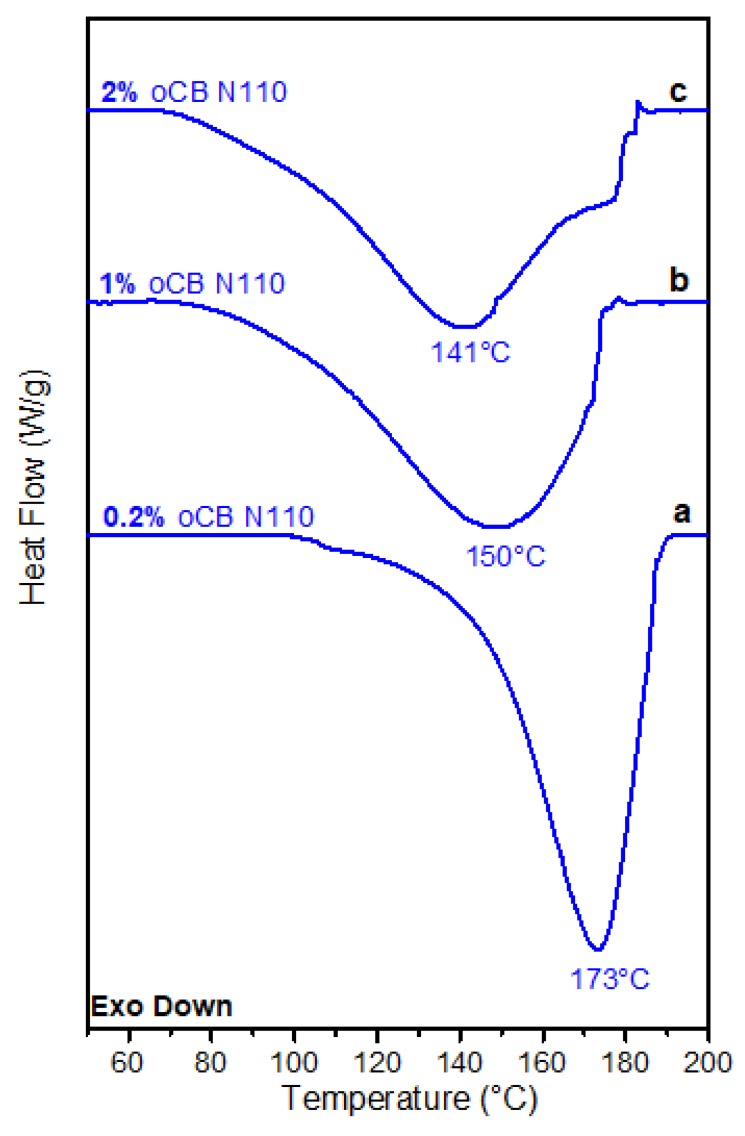
DSC scans at a heating rate of 10 °C/min for resorcinol/HMMM resins (for the weight ratio 1) and different amounts of oCB-N110: (**a**) 0.2 wt %; (**b**) 1.0 wt %; and (**c**) 2.0 wt %.

DSC scans of the resin with a weight ratio resorcinol/HMMM equal to 1 and with different amounts of oCB-N110, i.e., with the oCB sample with a higher O/C ratio, are shown in Figure 5. A remarkable influence of the carbon filler on resin crosslinking is clearly present also for a content of 1 wt %, with peak broadening and a shift of its maximum down to 150 °C (Figure 5b). The influence of the carbon filler on resin crosslinking becomes instead negligible for an oCB content as low as 0.2 wt % (Figure 5a).

Exothermic peak positions (*T*_peak_) and peak shifts (Δ*T* = *T*_peak_(unfilled) − *T*_peak_(filled)), as taken from DSC scans of Figure 4 and Figure 5, are collected in the 2^nd^ and 3^rd^ columns of Table 2.

Also informative are DSC scans of the same phenolic resin after isothermal curing at different temperatures. These kinds of scans allow obtaining quantitative evaluation of the degree of crosslinking (*α*) achieved by isothermal crystallization.

**Figure 6:**
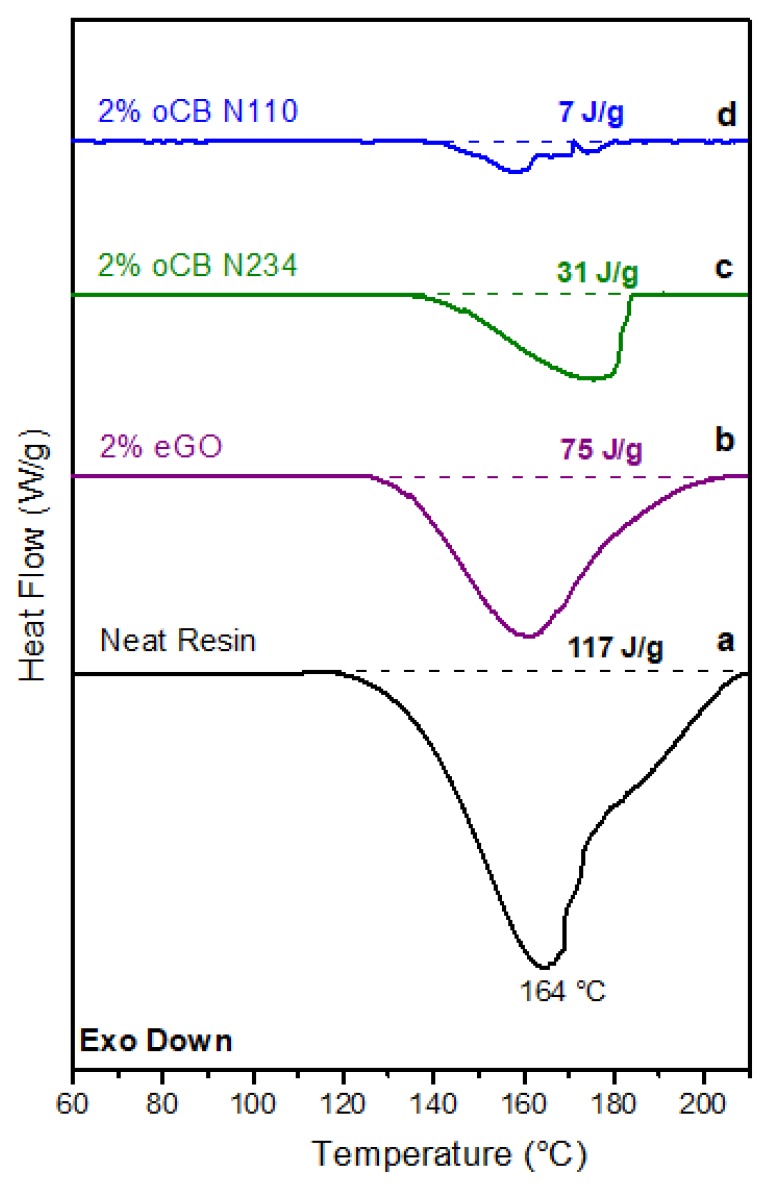
DSC scans at a heating rate of 10 °C/min for resorcinol/HMMM resins (for the weight ratio 1), after isothermal curing at 120 °C for 30 min: (**a**) without filler; (**b**–**d**) with 2 wt % of carbon filler: (**b**) eGO; (**c**) oCB-N234; and (**e**) oCB-N110.

DSC scans of resorcinol/HMMM, 1/1 resins after treatment at 120 °C for 30 min are shown in Figure 6, for the case of the neat resin as well as of resins containing 2 wt % of different oxidized carbon fillers. It is apparent that in all cases the enthalpy of the exothermic crosslinking peak is heavily reduced. In fact, for the neat resin, the enthalpy of the peak reduces from 270 J/g to 117 J/g, indicating that nearly 60% of the crosslinking reaction had already occurred at 120 °C. It is also apparent that the presence of the carbon nanofillers (mainly of the oxidized CB) strongly reduces the area of the exothermic peaks and hence of the residual crosslinking.

DSC scans like those of Figure 6 can be used to evaluate the degree of crosslinking (*α*) after the isothermal treatment, simply by evaluating the enthalpy of the residual exothermic peak (Δ*H*_res_) and applying the equation

*α* = (Δ*H*_unannealed_ − Δ*H*_res_)/Δ*H*_unannealed_, (2)

where Δ*H*_unannealed_ is the enthalpy of the exothermic peaks in dynamic scans. For our resin and for the considered resorcinol/HMMM 1/1 weight ratio, Δ*H*_unannealed_ = 270 J/g.

Residual crosslinking enthalpies after isothermal treatment at 120 °C for 30 min (Δ*H*_res_) as taken by DSC scans like those of Figure 6 are collected in the 4th column of Table 2. Degrees of crosslinking (*α*) after the considered isothermal treatment, as calculated from Δ*H*_res_ by using Equation (2), are collected in the 5th column of Table 2.

Scans of Figure 6 and data of Table 2 clearly show that the addition of 2 wt % of oCB-N110 is sufficient to achieve a nearly complete crosslinking already at 120 °C, while in the same conditions the neat resin has only reached a degree of crosslinking close to 60%.

The reported data clearly show that oxidized carbon black samples are much more efficient than graphene oxide as catalysts of crosslinking of the considered phenolic resin. The reported data also show that the higher catalytic activity is not due to the amount of oxidized groups. In fact, oCB N234, exhibiting an O/C weight ratio lower than for the considered eGO and similar sulfur amount (Table 1), leads to a much higher reduction of crosslinking peak temperature.

WAXD (Figure 2) and FTIR (Figure 3) characterizations of the used nanofillers suggest that the higher catalytic activity of oCB samples, with respect to GO samples, is possibly due to the higher exposure of the active oxidized groups on smaller and more defective graphene layers of oCB.

## 4. Conclusions

The influence of different oxidized graphite-based nanofillers (graphene oxide and oxidized carbon black) on the crosslinking reaction of a phenolic resin, constituted by resorcinol and hexa(methoxymethyl)melamine, was studied.

Curing reactions leading from low molecular mass compounds to crosslinked insoluble networks were studied by Differential Scanning Calorimetry by scans on as-mixed resins as well as on isothermally treated resins.

DSC scans show a catalytic activity of graphene oxide (eGO) on the considered crosslinking reaction, comparable to one already described in the literature for curing of benzoxazine. For instance, for an eGO content of 2 wt %, the exothermic crosslinking DSC peak (upon heating at 10 °C/min) is shifted from 173 °C to 167 °C.

The main result of this paper is that oxidized carbon black samples are much more effective than eGO as catalyst of the curing reaction. In fact, for the oCB sample exhibiting a comparable O/C ratio (0.58 vs. 0.62), for the same content in the resin (2 wt %), the crosslinking DSC peak is shifted nearly 25 °C, rather than 6 °C. This shift also becomes bigger (32 °C) by using an oCB sample with a higher O/C weight ratio (O/C = 0.83 by wt). The reported study also shows that, by using an oCB content of 2 wt %, an isothermal treatment at 120 °C is sufficient to get complete resin crosslinking, while in the same conditions the sample with eGO only reaches a degree of crosslinking close to 60%.

FTIR and WAXD characterizations of the used carbon nanofillers suggest that the higher catalytic activity of oCB, with respect to eGO, is possibly due to higher exposure of active oxidized groups on smaller and more defective graphene layers of oCB.

## Figures and Tables

**Table 1:** Elemental analysis of the obtained graphite oxide (GO), exfoliated graphene oxide (eGO) and of two oxidized carbon black (oCB) samples.

Sample	C (wt %)	H (wt %)	O (wt %)	S (wt %)	O/C
**GO**	56.1	1.2	39.8	2.7	0.71
**eGO**	59.4	0.6	37.1	2.6	0.62
**oCB N234**	60.3	1.7	35.0	2.8	0.58
**oCB N110**	50.3	2.3	41.7	5.4	0.83

**Table 2:** Peak positions (*T*_peak_) and peak shifts (Δ*T*) taken from dynamic DSC scans like those of Figure 3 and Figure 4. Residual crosslinking enthalpies after isothermal treatment at 120 °C for 30 min (Δ*H*_res_), as taken by DSC scans like those of Figure 6. Degree of crosslinking (*α*) after the considered isothermal treatment was calculated from Δ*H*_res_ by using Equation (2).

Sample	*T*_peak_ (°C)	Δ*T* (°C)	Δ*H*_res_ (J/g)	α (%)
**Neat Resin**	173	-	117	57
**oCB N110 0.2%**	173	0	108	60
**eGO 2%**	167	6	75	72
**oCB N110 1%**	150	23	40	85
**oCB N234 2%**	148	25	31	89
**oCB N110 2%**	141	32	7	97
